# Restoring Coal Mining-Affected Areas: The Missing Ecosystem Services

**DOI:** 10.3390/ijerph192114200

**Published:** 2022-10-30

**Authors:** Alicja Krzemień, Juan José Álvarez Fernández, Pedro Riesgo Fernández, Gregorio Fidalgo Valverde, Silverio Garcia-Cortes

**Affiliations:** 1 Department of Extraction Technologies, Rockburst and Risk Assessment, Central Mining Institute, 40166 Katowice, Poland; 2 School of Mining, Energy and Materials Engineering, University of Oviedo, 33004 Oviedo, Spain; 3 Polytechnic School of Mieres, University of Oviedo, 33600 Mieres, Spain

**Keywords:** coal mining, restoration, RECOVERY project, ecosystem services, valuation, people-centered ecologism, carbon allowances

## Abstract

Multi-criteria decision analysis and cost-benefit analysis, either individually or in combination, have been used as the preferred tools to develop ecosystem services valuation, presenting significant discrepancies and variations between the calculated values. To counteract this problem, a new framework was developed based on a hierarchical weighting of the non-provisioning ecosystem services, using biodiversity as the reference ecosystem service since it is the easiest to apprehend. Their monetisation was made using the average price of EU carbon dioxide emission allowances during 2019 and 2020, obtaining reasonable and comparable results in line with what was expected for the study region. However, the revised EU Emissions Trading System Directive, which will apply from 2021–2030, generated a price escalation of carbon allowances, making it necessary to adjust or rethink the proposed framework. To achieve this goal, the paper proposes the introduction of new vectors or “missing ecosystem services” to counterbalance efforts to eliminate carbon dioxide emissions without necessarily removing humans from the equation: welfare and human health. As the linkages regarding ecosystem health, ecological restoration and human health are not well known, only welfare was incorporated into the framework. The results were highly satisfactory, in line with what was expected for the study region and the ones obtained before the price escalation of carbon allowances that started in 2021.

## 1. Introduction

The paper “Valuation of ecosystem services based on EU carbon allowances—optimal recovery of a coal mining area” [1] proposed a new framework for evaluating ecosystem services, a concept that is often used to describe links between nature and the economy [2,3,4,5,6]. Up to now, multi-criteria decision analysis (MCDA) has been used as the preferred tool to integrate multiple values by assigning them a relative weight [7,8,9,10]. However, MCDA does not solve the problem caused by aggregating values obtained with different assessment methods [11,12,13]. Other tools, such as cost-benefit analysis (CBA), suffer from the same problem, either used individually [14] or in combination with MCDA [15]. Thus, despite all the studies and research, the values calculated for the different ecosystem services present significant discrepancies and variations between them [16,17].

The new framework initially follows the approaches by Spangenberg and Settele [18], who proposed valuing only real things, and Xie et al. [19], who calculated the value of ecosystem services based on the relative weight of each ecosystem service compared to a standard. It was based on a hierarchical weighting [20] of the non-provisioning ecosystem services of the study area, using biodiversity as the reference attribute/ecosystem service since it is the easiest to apprehend of all the ecosystem services. Instead of adding the values calculated using different methodologies, an initial weighting of the ecosystem services present in the study area is made, which will be later used to assign economic values.

To achieve this goal, first, it was necessary to define the scenarios that were considered feasible to undertake the ecosystem restoration of the study area and to determine all the ecosystem services that were going to be involved in the analysis. The purpose was to establish which ecosystem services are foreseen to be provided within the study area and to allow a quantification of the trade-offs among the provision of alternative ecosystem services [21,22,23]. Three scenarios were selected: (1) fibre, representing pine plantations to produce wood; (2) food, representing cows feeding for beef production; and (3) landscape, representing a broad-leaved forest similar to the existing ones in the area.

Once the scenarios were established, provisioning and non-provisioning ecosystem services considered representative of the different land covers were identified and quantified. The Common International Classification of Ecosystem Services (CICES) V5.1 [24] was the primary reference for selecting the various ecosystem services involved in the evaluation process. Ecosystem services were then given values according to their respective weight compared to the carbon sequestration ecosystem service, which is straightforward as there is a European market of carbon allowances. 

The results for the different proposed scenarios were very reasonable and in line with what was expected for the study region. Moreover, they had the same orders of magnitude. Therefore, they were comparable, giving confidence that the whole process was going in the right direction.

However, on July 2021, the EU adopted different legislative proposals addressing a climate neutrality goal by 2050 [25]. The revised EU Emissions Trading System Directive, which will apply from 2021–2030, generated a price escalation of the allowances for carbon emissions, making it necessary to adjust or rethink the proposed framework.

The most straightforward alternative would be to reconsider the hierarchical weighting previously developed, giving more significance (weight) to biodiversity compared to the rest of the ecosystem services. However, this alternative can be considered artificial and arbitrary, so exploring it does not seem advisable. 

Bellver Capella [26], in his article entitled “For a people-centred ecologism”, analyses the book written by the philosopher and jurist Ballesteros [27], proposing “an ecological and social personalism that not only cares for nature in general but also for human nature in particular, respecting the natural differences between human beings and fighting against the inequalities produced by political domination, economic exploitation or ludic violence”. 

To properly orient our relations with nature, “the inherent differences between human beings should be recognised while combating inequalities between them; in short, replacing the culture of discarding with the imperative of caring” and also stressing “the importance of the small and the local in the face of the logic of globalisation and scalability”. Ultimately, this involves cultivating a people-centred ecologism focused on caring for nature in general and human nature in particular, and recognising human differences to correctly orient our relation with nature.

Several authors have been working in this direction. Fischer et al. [28] argued that human lives must be reintegrated into the rest of the environment and that social-ecological restoration can help with this goal. Aronson et al. [29] previously explored the growing evidence of ecosystem dysfunction’s impact on human health, introducing a new dimension of ecological restoration and helping restore social capital, with positive effects on local communities and public health.

Following a similar trend from very different perspectives, Zhang et al. [30] analysed quantitatively the spatial relationship between landscape patterns and habitat quality. Zhou et al. [31] explored the relationship between recreational services and tourists’ well-being, while Plutino et al. [32] provided a framework for ecosystem services, regulating services, pollination and human health. Istanbuly et al. [33] addressed the relationship between socioeconomic variables and soil erosion in Polish catchments, while Cao et al. [34] evaluated the interaction between poverty reduction and ecosystem services in poverty counties, something essential to promote rural revitalisation strategy and the construction of an ecological civilisation. Finally, Sena and Ebi [35] highlighted the pressures and impacts of desertification, land degradation and drought on human health. 

The present paper will propose using new vectors or ecosystem services, referred to as “the missing ecosystem services”, to counterbalance efforts to eliminate carbon dioxide emissions without necessarily removing humans from the equation. To determine the validity of this assumption, the article will compare the results with what is expected according to the socio-economic situation of the study area and with the ones obtained before the price escalation of carbon allowances that started in 2021.

## 2. Materials and Methods

### 2.1. The RECOVERY Project

This paper presents the results of the RECOVERY project [36], titled “Recovery of degraded and transformed ecosystems in coal mining-affected areas”, which is funded by the Research Fund for Coal and Steel (RFCS), European Commission, under Contract number 847205, and also, by the Polish Ministry of Science and Higher Education, under Contract number 5036/FBWiS/2019/2.

The RECOVERY project focuses on land rehabilitation and ecological restoration of coal mining-affected areas, aiming to accelerate the recovery of degraded and transformed ecosystems into good ecosystems, evaluating the consequences of alternative courses of action by considering their capacity to provide multiple ecosystem services. Its approach is premised on the notion that a quantitative assessment of the trade-offs among the ecosystem services provided by alternative scenarios is necessary for sound decision-making. 

The project used six case studies for mapping, quantifying and evaluating the ecosystem services provision under different scenarios to first appraise other coal mining-affected areas, their ecosystems and ecosystem services. Two underground coal mines (Poland and Spain), an underground coal mine dumps complex with a thermally active mine dump (Czech Republic) and three opencast lignite mines (two in the Czech Republic and one in Germany), all of them in different stages of restoration.

The waste heaps of the underground coal mine of Figaredo, in Asturias, Spain, were the area selected for this paper. However, the new methodology was implemented in all the regions, with no limitations detected among the different case studies.

### 2.2. The Study Area

The Figaredo mine is a closed underground coal mine that became part of Hulleras del Norte S.A. S.M.E. (HUNOSA) in 1998. HUNOSA is a state-owned coal mining company based in Asturias, in the north of Spain. It owns one active underground coal mine, one washery, a power plant equipped with CO_2_ capture and many closed undergrounds and open pit facilities across Asturias.

Figaredo was one of the first mines in the Turón valley, dating back to 1867. Famous for having some of the best Spanish coking coal, the first coke batteries were built on this site in 1890. In the early 1930s, pits were made to mine coal, although projects were delayed because of the Spanish Civil War.

HUNOSA is undertaking the partial restoration of the Figaredo waste heaps nowadays. While sectors one and two have already been restored, sectors three and four are being re-mined and used to store waste. These terrains are an excellent opportunity to propose land rehabilitation and ecosystem restoration alternatives, covering an area of 67 hectares.

**Figure 1:**
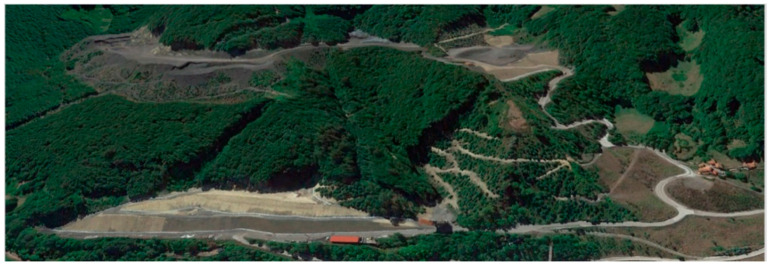
Figaredo mine and waste heaps.

Figure 1 shows the Figaredo mine area comprising the following areas: on the top left, sector four, which is being re-mined to recover coal; on the bottom left and in the recentre of the figure, sector two, which was already restored; on the top centre, sector three, which is being used to as a storage place for the newly generated waste; on the right and below the road, sector one, which underwent the self-restoration from the year 2009. 

It is critical to establish an ecosystem services context to determine the adequate but flexible boundaries of the area where the impact of the planned activities may produce changes. These changes can be found in the forms of land use, the monetary value of properties and the potential of ecosystem services. Considering the existing spatial connectivity in the surrounding area and the cohesion, an area of 238 ha was chosen. Then, the land cover of the site was mapped according to the CORINE Land cover [37,38] nomenclature, used as the reference.

### 2.3. Ecosystem Services Assessment

The next step was identifying land rehabilitation and ecosystem restoration scenarios that could be proposed in the Figaredo mining area. The following six alternatives were considered the most feasible by the Partners of RECOVERY, taking into consideration the Figaredo mine area features: (1) fibre: pine tree plantation for producing wood as a raw material; (2) food: cows reared for nutritional purposes; (3) landscape: the reconstruction of a broad-leaved forest similar to the ones already present in the region; (4) no restore: recolonisation by local vegetation with no restoration actions; (5) recreation: physical recreation area; and (6) solar: installation of renewable photovoltaic energy generation.

These alternatives were introduced in the Smic-Prob Expert [39,40] tool as the hypothesis for developing the scenario assessment. Smic-Prob Expert uses the cross-impact probability method to determine the most feasible scenarios.

The four scenarios with higher probability were Food (0.202), Fiber (0.176), Landscape + Recreation (0.135) and Landscape (0.118). Both Landscape + Recreation and Landscape alternatives have similar probabilities. Thus, a mixed scenario was proposed: it corresponds to the Landscape alternative as, simultaneously, it will be a physical recreation area where people can walk and observe nature around the area, as it will be a broad-leaved forest.

To promote the sprouting of herbaceous plants as well as to prevent an impact on the development of animal husbandry due to the pollution of the mining area, apart from water and soil analyses to control pollution, a 25 cm layer of topsoil was used to restore the waste heaps and to promote the sprout of herbaceous plants that were carefully selected to prevent pollutants’ transfer to the animals and maintain the sustainability and the quality of land [41,42].

Taking into account the selected scenarios and the land cover of the study area, an ecosystem service assessment was developed to choose those that can be considered relevant. Nine ecosystem services were finally selected, two provisioning and seven non-provisioning ecosystem services. The provisioning ecosystem services were fibre and food production. The non-provisioning ecosystem services were climate regulation (temperature and humidity), water flow regulation, erosion control, air purification, carbon sequestration and qualities of species or ecosystems (biodiversity).

**Figure 2:**
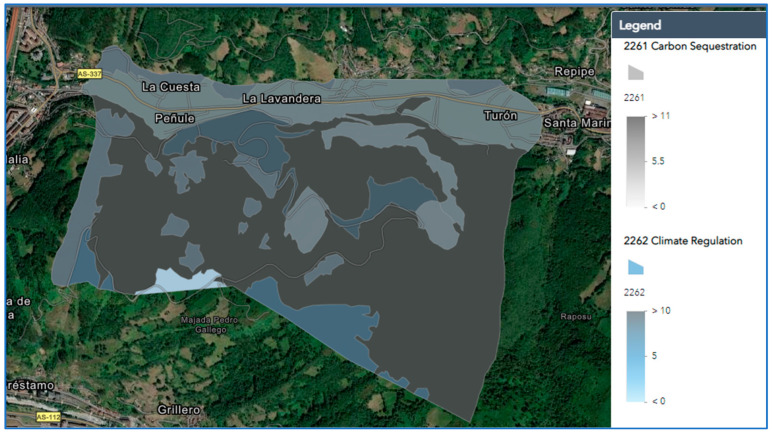
ArcGIS Online of the Figaredo mine area.

A geographic information system (GIS) web interface was then developed for the case study, allowing the construction of user-desired thematic information maps for viewing purposes and facilitating the subsequent identification and quantification of ecosystem services. Figure 2 shows the spatial data of the Figaredo mine area on the platform ArcGIS Online, with the following characteristics: attribute table by layer, polygon Geometry, attribute table data, polygon interactive context and existing land cover and ecosystem services tabs in the attribute table.

### 2.4. Economic Assessment

Once provisioning and non-provisioning ecosystem services were identified, it was time for their economic assessment. Provisioning ecosystem services were evaluated employing their net present value (NPV), using the investments and costs given by the market. Appropriate discount rates were proposed according to a specific classification of the non-provisioning services: (1) non-intensive natural goods production, such as familiar animal exploitation, tree plantations or agriculture; (2) intensive natural goods production, such as animal farms, forest exploitation or intensive agriculture; and (3) industrial goods production such as renewable energy or industrial facilities [43].

Non-provisioning ecosystem services were quantified before their economic estimation. For this, coefficients were used for each land cover type, obtained from different empirical studies [44]. These coefficients were normalised, and transformed into indices according to the work of Larondelle and Haase [45].

After quantifying the non-provisioning ecosystem services, it was time for their economic valuation or monetisation based on the hierarchical weighting done using biodiversity as the reference attribute/ecosystem service. Ecosystem services were given values according to their respective weight, compared to the carbon sequestration ecosystem service. Carbon sequestration assessment is straightforward since it is possible to use the EU Emissions Trading System [46] to determine the price of emitting a ton of carbon dioxide into the atmosphere. The average price of EU carbon dioxide emission allowances during 2019 and 2020 was used for this purpose: EUR 25/t [47].

## 3. Results

**Figure 3:**
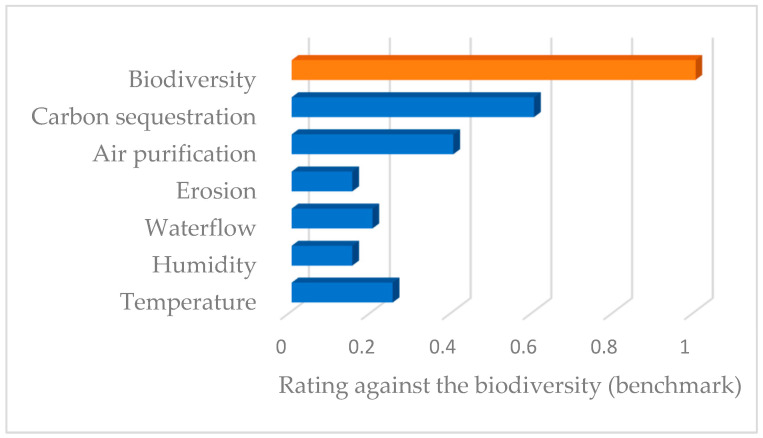
The weighting of non-provisioning ecosystem services with biodiversity as a benchmark (adapted from Krzemień et al. [1]).

Figure 3 presents the weighting of the non-provisioning ecosystem services of the study area using biodiversity as the reference ecosystem service, as it is the easiest to apprehend of all the ecosystem services involved in the case study. It was developed using the Delphi method with experts from Spain and Poland.

Using the average price of carbon allowances during 2019 and 2020 and assigning this value to a 100% contribution of carbon sequestration, according to the amount of CO_2_ that can be stored by the land cover with the highest value of above-ground carbon storage (broad-leaved forest), the value obtained is EUR 6267/ha [1]. It will be the value assigned to any 100% ecosystem service contribution. Thus, the maximum contribution of all the non-provisioning ecosystem services that are representative of the Figaredo mine area is presented in Table 1.

Table 2 presents the total value per ha of the considered scenarios. It adds the provisioning ecosystem services value calculated via the NPV plus the range contribution of non-provisioning ecosystem services based on the normalised coefficients obtained from different empirical studies that were transformed into indices for each land cover type [1]. A negative NPV is obtained in the Landscape scenario as there are only investment and maintenance costs related to the reconstruction of a broad-leaved forest similar to the ones already present in the region, as well as no income from provisioning ecosystem services such as wood. 

The three values have the same orders of magnitude; therefore, they are comparable, giving confidence that the whole process is going in the right direction. Although the scenario that offers the higher value is Food, the difference between Food and Fibre is only 3.3%. Thus, both scenarios could be considered plausible. The selection between them could be based on choosing the most easily achievable scenario (less investment needed) or producing profitability fastest. In the case of the Asturias region, it would undoubtedly be Food. Finally, the difference between Fiber and Landscape is 21%, and between Food and Landscape, 25%, values that can be considered significant to rule out the Landscape scenario.

## 4. Discussion

The EU Emissions Trading Scheme (EU ETS) is the first primary carbon market in the world and the largest. The EU ETS was introduced in 2005 and has reduced emissions by around 43% in the sectors covered by emissions trading

The EU ETS has undergone several changes since its introduction in 2005. The implementation was divided into four phases [46]. Phase 3 began in 2013 and lasted till 2020. Between 2019 and 2023, the number of allowances in reserve doubled to 24% of the allowances in circulation, significantly escalating Phase 3 prices in 2019 (from EUR 5/t to EUR 25/t). Finally, Phase 4, which started in 2021, coincided with a series of legislative proposals adopted by the European Commission on 14 July 2021, setting out how climate neutrality will be achieved by 2050. The proposals included an intermediate target of at least a 55% net reduction in greenhouse gas emissions by 2030, producing a vast price escalation to around EUR 80/t.

**Figure 4:**
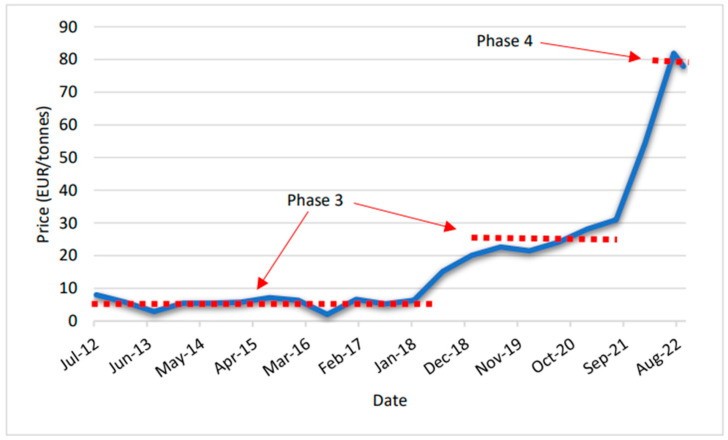
Prices of EU carbon permits from July 2012 (adapted from EMBER [47] and Trading Economics [48]). The dotted lines represent different periods of stable prices.

Figure 4 presents the prices of EU carbon permits from 2012 till September 2022, adapted from EMBER [47] and Trading Economics [48].

Taking into consideration the last level of prices of around EUR 80t, the new price for the highest value of above-ground carbon storage (broad-leaved forest) is EUR 20,056/ha. Table 3 presents the new maximum contribution of all the non-provisioning ecosystem services representative of the Figaredo mine area.

Table 4 presents the new total value per ha of the considered scenarios according to EU ETS Phase 4 prices.

We are, therefore, in a completely different situation, in which the Landscape scenario is the one that offers, without any doubt, the most outstanding value.

Although the evaluation of the proposed scenarios has radically changed, the study area’s situation has not changed at all. The socio-economic condition of the post-mining site, economically depressed due to the suppression of mining activity and with a high unemployment rate, cannot afford to drastically eliminate any form of economic activity for the sake of biodiversity. Moreover, in the surroundings of the Figaredo mine, biodiversity does not present a substantial problem.

As was mentioned before, a straightforward alternative would be to reconsider the hierarchical weighting previously developed, giving more significance to biodiversity. However, this alternative is artificial and arbitrary, so it is not the solution to the problem. Notwithstanding, the introduction of a new vector or vectors to counterbalance efforts to eliminate carbon dioxide emissions without necessarily removing humans from the equation then appears as a more promising alternative.

CICES V5.1, in its cultural ecosystem services, focuses solely and exclusively on the characteristics or features of living systems or on the elements of living systems of nature. The introduction of new ecosystem services focused on human nature to combat the inequalities between human beings pointed out by Ballesteros [18] should be proposed in the certainty that we are also an integrating part of biodiversity. They can be referred to as “the missing ecosystem services”. According to the authors working on reintegrating humans into the rest of the environment via socio-ecological restoration [19], human well-being could be considered the missing ecosystem service, which is defined as the state of being happy, healthy, or prosperous [49].

Nevertheless, Aronson [20] explored the evidence of ecosystem dysfunction’s impact on human health, introducing a new dimension to ecological restoration and helping to restore social capital. Thus, at least two new dimensions seem to appear: (1) welfare, the state of doing well, especially concerning good fortune, happiness, well-being or prosperity [50]; and (2) human health, the condition of being sound in body, mind or spirit [51].

Regarding these dimensions, the following two observations should be made. First, although welfare has the same meaning as well-being, as it can be considered more focused on prosperity, it seems to be the perfect word to illustrate the dimension relative to the wealth of the communities. Second, Aronson [52] explored the need to identify and strengthen the linkages regarding ecosystem health, ecological restoration and human health, as ecological restoration can enhance and become self-reinforcing for ecosystem health, human health and well-being. However, these linkages are not well known nor well defined, so introducing human health as an ecosystem service will have to wait to understand the concept and evaluate its contribution.

**Figure 5:**
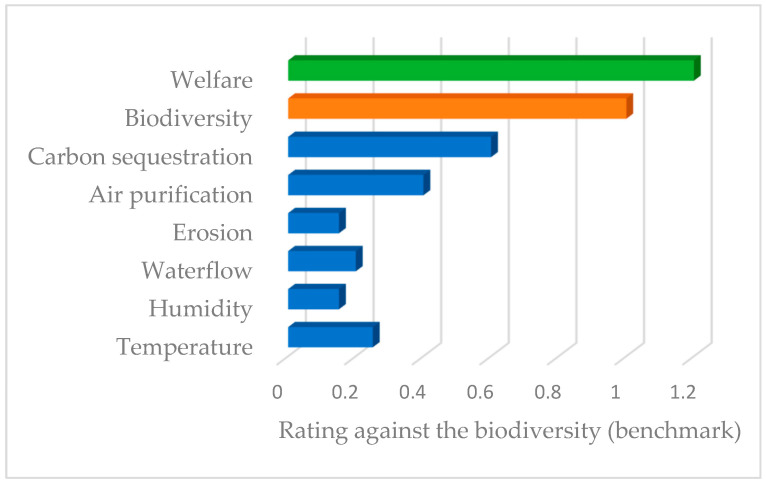
The weighting of non-provisioning ecosystem services with biodiversity as a benchmark, including the new ecosystem service of welfare.

According to this, the experts from Spain and Poland were asked to undergo the Delphi method regarding the weighting of the non-provisioning ecosystem services of the study area again, using biodiversity as the reference ecosystem service but introducing a new one: the welfare of the local community. The result obtained is presented in Figure 5.

Table 5 presents the maximum contribution of all the non-provisioning ecosystem services representative of the Figaredo mine area, including now welfare, which was evaluated at 20% over biodiversity.

Table 6 presents the new total value per ha of the considered scenarios according to EU ETS Phase 4 prices, including the consideration of the new ecosystem service: welfare.

After introducing welfare as a new ecosystem service, the scenario that offers the most significant value is again that of Food, an issue following the socio-economic situation of the study area due to the recession caused by the mine closure.

## 5. Conclusions

This article presents the novelty of introducing new ecosystem services, called “missing ecosystem services”, which are not covered by the Common International Classification of Ecosystem Services (CICES), based on the argument that there is a need to reintegrate human life with the rest of the environment and that socio-ecological restoration can play a crucial role in achieving this goal. In doing so, it aims to counteract the escalating price of carbon emission rights used in the valuation methodology by respecting the natural differences between human beings and fighting inequalities produced by political domination, economic exploitation or ludic violence.

The implementation of EU ETS Phase 4, which started in 2021, coincided with a series of legislative proposals adopted by the European Commission, setting out how climate neutrality will be achieved by 2050. These proposals included an intermediate target of at least a 55% net reduction in greenhouse gas emissions by 2030. This fact produced an increase in the carbon allowances price of approximately 312%, which led to a drastic change in the valuations of ecosystem services, starting with one of the most used valuations: carbon sequestration.

This has forced us to rethink the premise of ecosystem services valuation, being the most straightforward alternative to reconsider the significance of biodiversity. However, this alternative can only be considered artificial and arbitrary, so it cannot be regarded as a reasonable solution to the problem. Introducing a new vector or vectors to counterbalance the efforts to reduce greenhouse gas emissions without necessarily removing humans from the equation is a more promising alternative. Ultimately, this involves cultivating a people-centred ecologism focused on caring for nature in general and human nature in particular, recognising human differences to orient our relationship with nature correctly.

Once the solution to the problem was focused, it was necessary to decide which ecosystem services should be incorporated into the valuation set, as CICES V5.1 focuses solely and exclusively on the characteristics, features or elements of nature’s living systems. CICES V5.1 lacks a specific caring for human nature but its cultural and religious dimensions: human lives must be reintegrated into the rest of the environment, and social-ecological restoration should play a vital role.

According to these socio-ecological restoration trends, human well-being could be considered the missing ecosystem service. However, well-being is defined as the state of being happy, healthy, or prosperous. CICES V5.1 has a cultural ecosystem service related to the characteristics of living systems that enable aesthetic experiences which can be considered to be connected to happiness, as well as religion, entertainment, education and training. However, no ecosystem services are focused on human health and welfare, a word that this paper links with prosperity. Thus, human health and welfare could be considered the “missing ecosystem services”.

Finally, while welfare is quite an easy ecosystem service to apprehend, human health linkages with ecosystem health and ecological restoration are not well known nor well defined, so introducing this ecosystem service will have to wait to understand and evaluate its contribution fully.

Further research, apart from the one that is already taking place regarding human health linkages with ecosystem health and ecological restoration, should focus on the comparison between the proposed methodology and alternative calculation methods that are currently used, mainly based on multi-criteria decision analysis (MCDA) and cost-benefit analysis (CBA), either used individually or in combination.

## Figures and Tables

**Table 1:** Maximum contribution of ecosystem services per ha (adapted from Krzemień et al. [1]).

Ecosystem service	Weight	Value
Temperature	25%	EUR 1567
Waterflow	20%	EUR 1235
Erosion	15%	EUR 940
Air purification	40%	EUR 2507
Carbon sequestration	60%	EUR 3760
Humidity	15%	EUR 940
Biodiversity	100%	EUR 6267
Total		EUR 17,216

**Table 2:** The total value of the considered scenarios per ha (adapted from Krzemień et al. [1]).

Scenarios	Provisioning Ecosystem Services NPV	Non-Provisioning Ecosystem Services Range	Non-Provisioning Ecosystem Services Value	Total Value
Landscape	EUR -5486	0.93	EUR 16,011	EUR 10,525
Fibre	EUR 2386	0.60	EUR 10,330	EUR 12,716
Food	EUR 3323	0.57	EUR 9813	EUR 13,136

**Table 3:** New maximum contribution of ecosystem services per ha according to EU ETS Phase 4.

Ecosystem Service	Weight	Value
Temperature	25%	EUR 5014
Waterflow	20%	EUR 4011
Erosion	15%	EUR 3008
Air purification	40%	EUR 8022
Carbon sequestration	60%	EUR 12,034
Humidity	15%	EUR 3008
Biodiversity	100%	EUR 20,056
Total		EUR 55,154

**Table 4:** The new total value of the considered scenarios per ha according to EU ETS Phase 4.

Scenarios	Provisioning Ecosystem Services NPV	Non-Provisioning Ecosystem Services Range	Non-Provisioning Ecosystem Services Value	Total Value
Landscape	EUR 5486	0.93	EUR 51,293	EUR 45,807
Fibre	EUR 2386	0.60	EUR 33,092	EUR 35,478
Food	EUR 3323	0.57	EUR 31,438	EUR 34,761

**Table 5:** New maximum contribution of ecosystem services per ha according to EU ETS Phase 4, including welfare.

Ecosystem Service	Weight	Value
Temperature	25%	EUR 5014
Waterflow	20%	EUR 4011
Erosion	15%	EUR 3008
Air purification	40%	EUR 8022
Carbon sequestration	60%	EUR 12,034
Humidity	15%	EUR 3008
Biodiversity	100%	EUR 20,056
Welfare	120%	EUR 24,067
Total		EUR 79,221

**Table 6:** The new total value of the considered scenarios per ha according to EU ETS Phase 4, including the welfare ecosystem service.

Scenarios	Provisioning Ecosystem Services NPV	Non-Provisioning Ecosystem Services Range	Non-Provisioning Ecosystem Services Value	Total Value
Landscape	EUR -5486	0.65	EUR 51,293	EUR 45,807
Fibre	EUR 2386	0.66	EUR 52,346	EUR 54,732
Food	EUR 3323	0.70	EUR 55,504	EUR 58,829

## Data Availability

The data supporting reported results can be found at https://recoveryproject.uniovi.es/ (accessed on 7 October 2022).
